# Collagen from the Marine Sponges *Axinella cannabina* and *Suberites carnosus*: Isolation and Morphological, Biochemical, and Biophysical Characterization

**DOI:** 10.3390/md15060152

**Published:** 2017-05-29

**Authors:** Leto-Aikaterini Tziveleka, Efstathia Ioannou, Dimitris Tsiourvas, Panagiotis Berillis, Evangelia Foufa, Vassilios Roussis

**Affiliations:** 1 Department of Pharmacognosy and Chemistry of Natural Products, Faculty of Pharmacy, National and Kapodistrian University of Athens, Panepistimiopolis Zografou, Athens 15771, Greece; ltziveleka@pharm.uoa.gr (L.-A.T.); eioannou@pharm.uoa.gr (E.I.); foufa_e@hotmail.com (E.F.); 2 Institute of Nanosciences and Nanotechnology, NCSR “Demokritos”, Aghia Paraskevi 15310, Attiki, Greece; d.tsiourvas@inn.demokritos.gr; 3 Department of Ichthyology and Aquatic Environment, School of Agricultural Sciences, University of Thessaly, Fytoko Str., Nea Ionia 38445, Magnesia, Greece; pveril@apae.uth.gr

**Keywords:** *Axinella cannabina*, *Suberites carnosus*, sponges, marine collagen

## Abstract

In search of alternative and safer sources of collagen for biomedical applications, the marine demosponges *Axinella cannabina* and *Suberites carnosus*, collected from the Aegean and the Ionian Seas, respectively, were comparatively studied for their insoluble collagen, intercellular collagen, and spongin-like collagen content. The isolated collagenous materials were morphologically, physicochemically, and biophysically characterized. Using scanning electron microscopy and transmission electron microscopy the fibrous morphology of the isolated collagens was confirmed, whereas the amino acid analysis, in conjunction with infrared spectroscopy studies, verified the characteristic for the collagen amino acid profile and its secondary structure. Furthermore, the isoelectric point and thermal behavior were determined by titration and differential scanning calorimetry, in combination with circular dichroism spectroscopic studies, respectively.

## 1. Introduction

Collagen is an ubiquitous high molecular weight fibrous protein occurring in both invertebrate and vertebrate organisms, existing in more than 20 different types depending on its role in distinct tissues [1,2]. Its polypeptide chains are organized in a unique structure, in which three α-helices are intertwined forming a characteristic right-handed triple helix. These peptides are rich in glycine, proline, and hydroxyproline amino acids, all being crucial for the formation of the helical configuration [3].

Due to its high biocompatibility and biodegradability, collagen finds a plethora of applications, primarily in the sectors of cosmetics, pharmaceuticals, and medical care products [4,5]. Additionally, gelatin, the denatured form of collagen obtained by its partial hydrolysis, is used as an additive in the food processing industry and in nutraceuticals [6]. Its intrinsic low immunogenicity renders this natural biopolymer an ideal material for bone grafting, tissue regeneration, and construction of artificial skin [7,8]. Collagen destined for industrial use originates mainly from bovine and porcine sources, via an acid hydrolysis-based procedure [9]. Incidences of allergic reactions and connective tissue disorders, such as arthritis and lupus, as well as bovine spongiform encephalopathy and transmissible spongiform encephalopathy [10], have led to the reconsideration of cattle as the main source for collagen production. Furthermore, porcine collagen is prohibited for the Muslim and Jewish communities due to religious restrictions. Taking into account these two limitations, an alternative and safer source is currently actively sought.

Nowadays, collagen of marine origin as an alternative to mammalian sources is gaining ground, especially since the employment of recombinant technology is excluded due to its high cost [11,12]. Since collagen is the major constituent of the extracellular matrices of all metazoans, sponges are considered as one of the most promising sources [13,14,15]. Sponges, belonging in the phylum Porifera, composed of a mass of cells forming a porous skeleton made of organic (collagen fibers and/or spongin, especially in the case of the class Demospongiae) and inorganic (spicules) components, are the most primitive among multicellular animals (Metazoa) [16,17]. Marine sponges have been proven an inexhaustible source of secondary metabolites exhibiting diverse pharmacological properties [18,19,20,21]. In addition to these, macromolecules have gained interest since such biopolymers possess a wide range of bioactivities that can find applications in the biomedical sector. Collagen has been isolated from different marine sponges, e.g., *Spongia graminea*, *Microciona prolifera*, *Haliclona oculata* [22], *Hippospongia communis*, *Cacospongia scalaris* [23], *Geodia cydonium* [24] *Chondrosia reniformis* [25,26], and various *Ircinia* species [27], and in certain cases has shown high potency in tissue regeneration [28]. Although the importance of marine collagen has been recognized, only a few thorough investigations on marine sponges have so far been reported [25,27,29], probably due to its characteristic insolubility and mineralization, which cause difficulties in its isolation and characterization [30,31].

In the present study we report, for the first time, the isolation and characterization of collagens from the marine demosponges *Axinella cannabina* (Axenillidae) and *Suberites carnosus* (Suberitidae). By employing two different experimental approaches, the insoluble collagen (InSC), intercellular collagen (ICC), and spongin-like collagen (SlC) were obtained. The morphology of these collagens was analyzed by scanning electron microscopy (SEM), and their fibril formation and characteristic band periodicity was studied by transmission electron microscopy (TEM). Their secondary structure was evaluated based on their FT-IR spectra, while the amino acid composition of the ICCs was also determined. The thermal behavior of the ICCs was investigated by differential scanning calorimetry (DSC) and circular dichroism (CD) analyses.

## 2. Results

### 2.1. Isolation of Sponge Collagen

Two different procedures were used for the isolation of collagens from the demosponges *A. cannabina* and *S. carnosus*. The first method was initially introduced for the isolation of insoluble collagen (InSC) from *G. cydonium* [24] and *C. reniformis* [26] by employing an alkaline, both denaturing and reducing, homogenization buffer affording collagen in high yield. The second one utilizes a trypsin-containing extraction buffer, known to destroy the interfibrillar matrix and, therefore, releasing the collagen fibrils (ICC) [22,23]. After exhaustive water extraction, the remaining debris generally comprises the spongin/spongin-like collagen. In our case, since the specific sponges are deprived of spongin, the isolated samples are considered to contain spongin-like collagen (SlC).

The InSCs obtained by the application of the first method corresponded to 12.6% and 5.0% of the sponges’ dry weight for *A. cannabina* and *S. carnosus*, respectively (Table 1). Application of the second method resulted in the isolation of ICC and SlC, leveling to 3.0% and 42.8% dry weight for *A. cannabina* and 1.9% and 21.8% dry weight for *S. carnosus*, respectively (Table 1). The percentages found for the ICC yield are in accordance with previously-reported results for *Hippospongia gossipina* [32].

The siliceous or calcareous sponges are characterized by a large number of inorganic spicules, which, in *Haliclona* and *Microciona*, are bound together by spongin [22]. In order to remove the expected siliceous spicules in the InSC, the samples were treated with an HF solution for 20 min at room temperature to obtain spicule-free insoluble collagen (SF-InSC). The spicules accounted for 32% and 49% (*w*/*w*) of the sponges’ InSCs from *A. cannabina* and *S. carnosus*, respectively.

### 2.2. Examination of Surface Morphology

**Figure 1:**
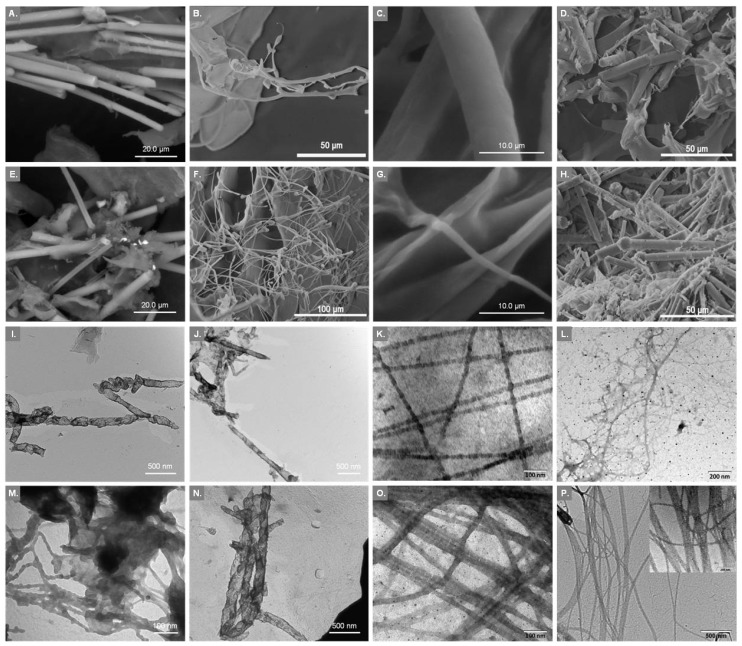
SEM micrographs of insoluble collagen (InSC; **A**,**E**), intercellular collagen (ICC; **B**,**C**,**F**,**G**), and spongin-like collagen (SlC; **D**,**H**) from *A. cannabina* (row 1) and *S. carnosus* (row 2), respectively. TEM micrographs of insoluble collagen before (InSC; **I**,**M**) and after (SF-InSC; **J**,**N**) spicule removal, intercellular collagen (ICC; **K**,**O**) and spongin-like collagen (SlC; **L**,**P**) from *A. cannabina* (row 3) and *S. carnosus* (row 4), respectively.

The collagenous nature of the isolated materials was investigated by SEM and TEM. Overall, the microscopically-observed structures (Figure 1) were similar to those already reported for collagen isolated from other sponges [25,26,27,29]. Figure 1A,E show the microstructure of the InSCs from *A. cannabina* and *S. carnosus*, respectively, as observed by SEM analysis. Smoothly wrinkled and folded sheets were evident. Additionally, the SEM pictures revealed that both sponges possess significant amount of spicules embedded in the very thin and soft sheet-like collagenous structure [22]. After removal of the spicules, the SF-InSCs appeared more as an amorphous matrix, while TEM depicted (Figure 1J,N) the collagenous material as appearing transparent, resembling those obtained before treatment with HF (Figure 1I,M). The complete removal of spicules was also confirmed by SEM (data not shown), where no silicate spicules were observed.

In the case of the SlCs, analogous structures were visible. In particular, siliceous spicules, known to support the sponges and provide defense against predation, were also detected (Figure 1D,H). On the other hand, ICCs presented the typical striation and sheet-like appearance of collagen fibers (Figure 1B,C,F,G), which conclusively proved the collagenous nature of the materials. Specifically, the ICCs from both sponges were observed as threads of various diameters along with the collagen sheets which is a combination of several collagen fibrils and fibers that are bundled together to form a fibril network and a dense pleated sheet-like structure. Sheets were smoothly wrinkled and folded, and appeared as very thin and soft (Figure 1C,G). Pleating of the sheets was visible at a magnification of 5000×.

The collagenous nature of the ICCs of *A. cannabina* and *S. carnosus* was further proved by TEM studies (Figure 1K,O). The obtained micrographs revealed the existence of filaments composed of striated collagen fibrils with repeated band periodicity, a characteristic feature of collagens, as observed earlier for sponge collagen fibrils [16]. Collagen fibrils were organized into bundles, while fibrils became aligned laterally in an ordered way, or curled into bundles consisting of up to 20 fibrils [24]. The individual fibrils displayed a visible, regular transverse banding pattern of about 300 Å periodicity (313 and 288 Å for *A. cannabina* and *S. carnosus*, respectively). These banding patterns are in accordance with the one reported for collagen fibrils isolated from *C. reniformis* [25,26]. The bundles revealed remarkable uniformity in the diameter of their constitutive fibrils (Table 2) with an average of 187 and 199 Å for *A. cannabina* and *S. carnosus*, respectively, in accordance with previously-reported data for other sponges [25,29].

The recorded TEM micrographs for the InSCs (Figure 1I,M) and the SlCs (Figure 1L,P) samples did not present a characteristic pattern. However, in the case of the SlC isolated from *S. carnosus* an area with clearly-striated collagen was detected (Figure 1P insert), most likely due to the nature of the preparation, composed of a mixture of ICC and SlC, also previously reported by Gross and coworkers [22]. The lack of a clear banding pattern might be attributed to the isolation, under the described conditions, of dominating collagenous structures presenting common characteristics with basement membrane (type IV) collagen. Transparent sheets of collagenous material were also previously observed for irciniid collagens, attributed to the non-fibrillar basement-type resembling collagens [27].

### 2.3. Infrared Spectroscopic Analysis

In the IR spectra of the isolated collagenous materials, all characteristic absorption bands of amides I, II, and III, as well as amides A and B (Table 3), indicative of the secondary structure of the different materials [33], were observed. The amide A absorption band, associated with the hydrogen-bonded N-H stretching vibration [34], was observed at lower frequencies (3279–3294 cm^−1^), as opposed to the free N-H stretching vibration that appears in the range of 3400–3440 cm^−1^. This peak is shifted at lower frequencies than the ones reported for the collagen of the marine sponge *C. reniformis* [25] and the calf skin type I collagen, indicating that the N-H group is involved in extensive hydrogen bonding, which stabilizes the helical structure of collagen [34,35,36]. On the other hand, the amide B band, related to the asymmetrical stretch of CH_2_ and NH_3_^+^ [36,37] remained relatively constant (~2924 cm^−1^), pointing to the absence of major differences in the lysine content in all of the examined samples [37].

**Figure 2:**
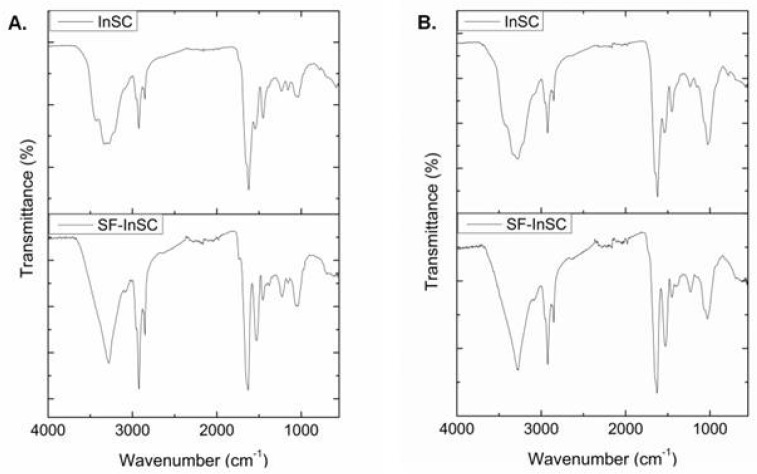
IR spectra of insoluble collagen before (InSC; upper) and after (SF-InSC; lower) spicule removal isolated from *A. cannabina* (**A**) and *S. carnosus* (**B**).

The amide I band, mainly associated with the C=O stretching vibration coupled with the N-H bending vibration along the polypeptide backbone or with hydrogen bonding coupled with COO^−^, C-N stretching, and CCN deformation, is the most intense band in proteins and, therefore, the most sensitive and useful marker for the analysis of the secondary structure of proteins with IR spectroscopy [38]. Normally resonating in the range of 1600–1700 cm^−1^ [39,40], bands around 1630 cm^−1^ indicate imide residues, and bands around 1660 and 1675 cm^−1^ are assigned to intermolecular crosslinks and b-turns, respectively [38]. In our samples, the amide I peaks are shifted to lower frequencies, indicative of higher hydrogen bonding potential [37], less intermolecular cross-linking, and decreased molecular order [39]. The lowest frequencies were observed in both InSCs, before and after treatment with HF, whereas the frequencies increased in the cases of the SlC and ICC samples, concomitantly to the molecular order increase. Additionally, the amide II band, associated with the N-H bending vibration coupled with the C-N stretching vibration, was also shifted to lower frequencies (1527–1547 cm^−1^), indicative of the involvement of the N-H group in hydrogen bonding [35]. Finally, the amide III band, attributed to the C-N stretching vibration in combination with the N-H deformation, is considered as the collagen fingerprint because it is accredited to the characteristic collagen repeating tripeptide (Gly-X-Y) [38]. Furthermore, in the IR spectra of the isolated collagenous materials, additional bands at about 1030 cm^−1^ appeared, mostly attributed to C-O vibrations due to the presence of carbohydrates [25,41]. In the case of the SF-InSCs, in the recorded IR spectra (Figure 2) a less intense peak at ~1030 cm^−1^ appeared, possibly corresponding also to the Si-O-Si asymmetric bond stretching vibration, known to absorb in the range of 1030–1100 cm^−1^.

The absorption intensity ratio between the amide III band (1238 cm^−1^) and the band at approximately 1450 cm^−1^ was 0.88 and 0.89 for the ICCs of *A. cannabina* and *S. carnosus*, respectively, indicating that the triple helix has been adequately preserved during the isolation procedure [34,36,37,40]. Generally, a ratio of approximately 1 indicates that the triple helical structure of collagen is intact [42]. In the case of the InSCs, this ratio is low for both sponges (~0.7), indicating that the triple-helical structure might be slightly affected during the extraction procedure. It was shown earlier that this ratio might be lower when the collagen triple helix is affected by cleavage of telopeptides through pepsin digestion [36]. Moreover, upon treatment for the removal of spicules, the absorption intensity ratio between amide III band and the band approximately at 1450 cm^−1^ increased to 0.96 and 0.94 for *A. cannabina* and *S. carnosus*, respectively, demonstrating the removal of other impurities.

### 2.4. Isoelectric Point Determination

**Figure 3:**
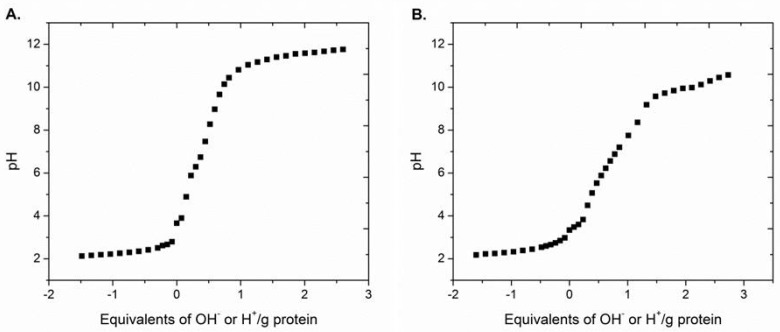
Titration curves of insoluble collagen after spicules removal (SF-InSC) isolated from *A. cannabina* (**A**) and *S. carnosus* (**B**).

The SF-InSCs were subjected to titration for the determination of the acid-base properties and the isoelectric point. The titration curves are shown in Figure 3. The pH of freshly-prepared InSC dispersions were 3.48 and 3.54 for *A. cannabina* and *S. carnosus*, respectively. After the HF treatment, the pH of the SF-InSC dispersions were slightly altered (3.66 and 3.34 for *A. cannabina* and *S. carnosus*, respectively). These values are lower than those reported for *C. reniformis* [26], probably due to the higher content in acidic amino acids (aspartic acid or glutamic acid), as also supported by the high contents of Asx and Glx found in both sponges from the amino acid content analysis (Table 4). The isoelectric point was calculated to be approximately 6.7 and 6.3 for *A. cannabina* and *S. carnosus*, respectively. These results are in agreement with previously-reported data determining the isoelectric point of insoluble collagen at pH values around 7 [26].

### 2.5. Amino Acid Profile

The amino acid composition of collagen is one of the key factors affecting its properties. Therefore, the amino acid profile of the ICCs from both sponges was determined (Table 4). Their composition was analogous to that described for the sponges *G. cydonium*, *C. reniformis*, and *I. variabilis* [23,24,25,26]. Glycine (Gly) was found to be the major amino acid in both ICCs with 257 and 295 residues/1000 residues for *A. cannabina* and *S. carnosus*, respectively. This result is in accordance with the Gly-X-Y amino acid model in which Gly occurs in every third position. Relatively high contents of aspartic acid (Asx; 100 and 94 residues/1000 residues), glutamic acid (Glx; 82 and 84 residues/1000 residues), alanine (Ala; 72 and 89 residues/1000 residues), and proline (Pro; 58 and 56 residues/1000 residues) were observed for *A. cannabina* and *S. carnosus*, respectively. Both ICCs presented the characteristic high threonine (Thr) and serine (Ser) content (approximately 6% each) and low lysine (Lys) and hydroxylysine (Hyl) content, previously reported for *Ircinia* [23]. Low Hyl content (5–6 residues/1000 residues) has also been reported for acid- and pepsin-soluble collagens isolated from shark skin [39]. Moreover, the sum of Thr and Ser of both sponges’ ICCs is similar to that of collagens reported for lower vertebrates and invertebrates. Additionally, no differences in the Lys content of the two different ICCs were observed, as already indicated from the same absorption bands in the IR spectra at 2922 cm^−1^ attributed to amide B (Table 3) [37].

Compared to *S. carnosus*, ICC from *A. cannabina* contained higher amounts of methionine (Met), phenylalanine (Phe), leucine (Leu), and isoleucine (Ile), but lower amounts of Gly, Ala, and hydroxyproline (Hyp). The percentage of the remaining amino acids is in relatively good agreement to the above-mentioned studies, especially the amounts of Asx, Glx, Pro, His, and Ala. The number of sulfur-containing Met residues was significantly higher in the ICCs of both sponges (Table 4), as compared to collagen from porcine dermis (6 residues/1000 residues) [43].

Nevertheless, the overall percentages of Hyp were lower than those reported for other sponges [22]. Imino acids are involved in hydrogen bonding, therefore affecting the stability of the collagen triple helix and its thermal behavior [37,39,44]. The imino acid content value is usually lower in marine collagens in comparison to mammalian collagens, resulting in a lower thermal denaturation temperature [33].

The ICCs from both sponges contained approximately 12 tyrosine (Tyr) residues per collagen molecule, indicating that their nonhelical telopeptides, where all of the Tyr residues are located, were intact [45]. The reduced values for Gly, Hyp, and Hyl can also be attributed to the existence of glycoproteinaceous impurities, known to be strongly associated with collagen [46].

### 2.6. Thermal Behaviour

**Figure 4:**
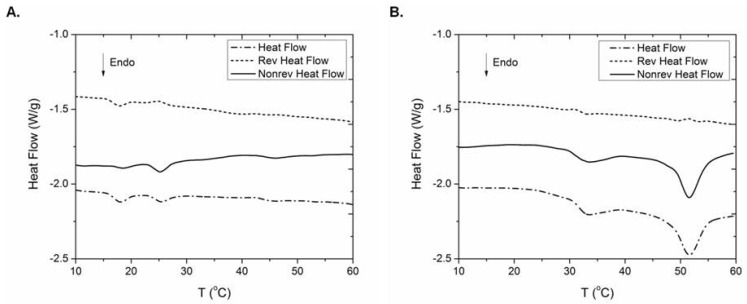
Temperature modulated DSC data of intercellular collagen (ICC) isolated from *A. cannabina* (**A**) and *S. carnosus* (**B**). The total (---), non-reversing (―) and reversing heat (-∙-) flows are presented (curves are shifted vertically for clarity).

It is well established [47] that upon increasing temperature, thermal denaturation of collagen is taking place, during which hydrogen bonds break and helices unfold, leading to the formation of collagen coils. This process is accompanied with appreciable heat absorption and can, therefore, be monitored with DSC. Indeed, the DSC curves of the hydrated collagen samples (Figure 4) clearly indicate two major endothermic peaks. Previous DSC studies also revealed collagen’s bimodal transition and concluded that the higher temperature peak was due to the helix-coil transition of collagen (denaturation of collagen), while the lower temperature peak originated from the breaking of the hydrogen bonds between collagen molecules or the defibrillation of the solubilized collagen fibrils [48,49]. This is attributed to the fact that the inter-triple helix hydrogen bonds responsible for the fibrillation are easier to break than the intra-triple hydrogen bonds that are responsible for helix formation [48].

In the present study, the thermal behavior of the ICCs isolated from *A. cannabina* and *S. carnosus* were monitored after removal of the entangled glycoconjugates [46]. The yield of the described procedure was 38% and 46% (*w*/*w*) for *A. cannabina* and *S. carnosus*, respectively. The low endothermic transition had its peak maximum transition temperature (T_max_) at 25.4 °C (Δ*H* value 1.27 J g^−1^) and 32.9 °C (Δ*H* value 5.74 J g^−1^) for the ICCs from *A. cannabina* and *S. carnosus*, respectively (Figure 4). The high temperature endothermic peak had a T_max_ of 44.6 °C (Δ*H* value 0.37 J g^−1^) and 51.6 °C (Δ*H* value 17.65 J g^−1^) for the *A. cannabina* and *S. carnosus* ICCs, respectively (Figure 4). As is clearly evident from the examination of both reversing and non-reversing components of the thermograms, the total heat flow for the thermal denaturation of collagen involves a significant non-reversing component, while the reversing component is negligible. This is in line with previous studies that showed that collagen denaturation endotherms in fibers and in basement membranes are governed by an irreversible rate process [50,51] and not by equilibrium thermodynamics, as previously hypothesized. Given the irreversibility of the process within the time frame of temperature modulation (60 s), the transitions are registered as essentially a non-reversing event in temperature-modulated differential scanning calorimetry (TMDSC), although, in general, unfolding of proteins is a complex phenomenon that encompasses both reversible and irreversible steps [52].

A rather low T_max_ value, as that observed for the ICC from *A. cannabina*, was reported earlier for collagen isolated from edible jellyfish (26.0 °C) [53]. On the other hand, T_max_ values around 31 to 33 °C, as that measured for the ICC from *S. carnosus*, have been observed for an array of collagens isolated from tropical fish [34,54]. Moreover, the ICC from *S. carnosus* exhibited a higher Δ*H* value (5.74 J g^−1^) than that of *A. cannabina* (1.27 J g^−1^). It is widely accepted that T_max_ is directly correlated with imino acid content, body temperature of the specimen, and environmental temperature [55,56], whereas the enthalpy change (Δ*H*) can be influenced by molecular stability, directly correlated with the amino acid sequence in collagen.

In our case, the ICCs from both sponges contain low amount of iminoacids (Table 4) in comparison to that of terrestrial organisms (approximately 200 residues/1000 residues), with the ICC from *A. cannabina* displaying the lowest content (96 residues/1000 residues). The observed difference between the T_max_ of the ICC samples from the two sponges could, therefore, be attributed to the imino acid content difference, and especially to the Hyp content difference. This phenomenon might also be related to the superior stability of the ICC from *S. carnosus*, due to the high content of the Gly-X-Y sequence, as confirmed by the elevated percentage of Gly (17.9% vs. 15.0% *w*/*w* for *S. carnosus* and *A. cannabina*, respectively). This, in agreement with previous reports [33,57], might be an additional justification for the high value of T_max_ despite the low amount of imino acids. Additionally, as previously reported [58], the high Asp (pK ≈ 3.9) and Glu (pK ≈ 4.3) content can contribute to ion pair formation with the basic residues at neutral pH, resulting in increased stability, which might partially compensate for the decreased stability deriving from the low Hyp content (Table 4) [59]. Another possible reason might be the intensely-localized sulfur bonding interactions associated with the higher Met content [43].

Finally, an additional low temperature endothermic peak (T_max_ = 17.9 °C, Δ*H* = 1.65 J g^−1^) was observed in the case of the ICC from *A. cannabina*. In contrast to the previous transitions discussed, the examination of both the reversing and non-reversing components of this specific transition suggests that this process is, to a great extent, reversible. Taking into consideration that during the denaturation of small proteins (for instance lysozyme) [60] the reversible unfolding has the largest contribution, whereas the irreversible process still remains well detectable, we tentatively ascribe this low temperature transition to the denaturation of small molecular weight collagen species that are present in this sample.

**Figure 5:**
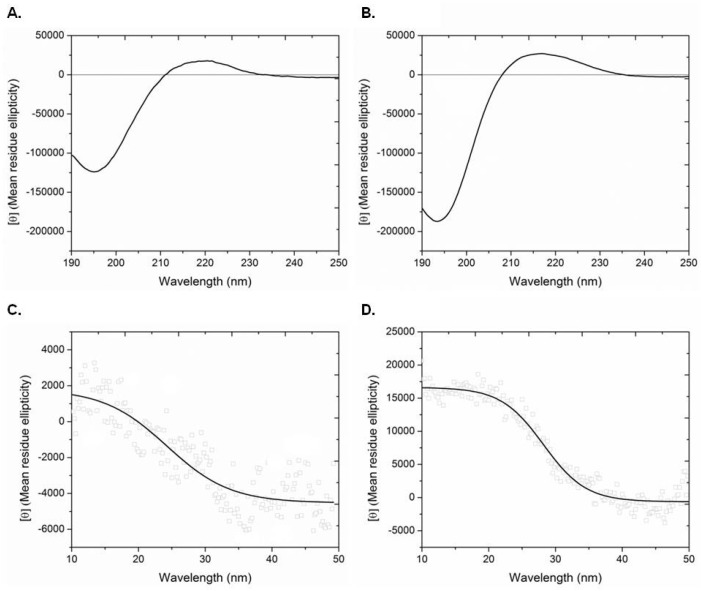
CD spectra in the region of 190–250 nm (recorded at 20 °C) and temperature effect on the CD spectra at 221 nm of intercellular collagen (ICC) isolated from *A. cannabina* ((**A**) and (**C**), respectively) and *S. carnosus* ((**B**) and (**D**), respectively).

The CD spectra of the ICCs from the two sponges in the region of 190 to 250 nm are depicted in Figure 5A,B. Both samples showed a rotatory maximum at about 221 nm, a minimum at 193–196 nm, and a consistent crossover point (zero rotation) at about 212 nm. These spectral characteristics are typical of a collagen triple-helix structure [61,62,63]. The corresponding mean residue ellipticities, [*θ*]_221_, as a function of temperature, are shown in Figure 5C,D. The [*θ*]_221_ values decreased with temperature due to decomposition of the collagen triple helical structure, and indicated denaturation temperatures of 24.3 °C and 28.2 °C for the ICCs from *A. cannabina* and *S. carnosus*, respectively, in good agreement with the obtained results from the conducted DSC studies.

It has been earlier shown that thermal denaturation temperature of collagens from different sources correlates directly with the imino acid (Pro and Hyp) content [43,64]. Actually, higher imino acid content facilitates intra- and intermolecular crosslinking resulting in a more stable triple helical structure of the collagen molecule [44]. A good linear correlation was observed earlier when measured denaturation temperatures were plotted against the corresponding numbers of Hyp residues, this effect being less pronounced with respect to the Pro content [43]. The amino acid composition analysis of the investigated sponges (Table 4) confirms the above observations, since *A. cannabina* presents a lower Hyp, but equal Pro, content as compared to *S. carnosus* resulting, therefore, in a concurrently-reduced T_d_. Interestingly, cold-water fish collagens have low T_d_ since their imino acid contents are very low [65], in contrast to the T_d_ of skin collagen of terrestrial mammals which are 37 °C and 40.8 °C, respectively [43], both possessing high imino acid content.

## 3. Discussion

The presence of collagen in freshwater, as well as marine sponges, was unequivocally established more than 50 years ago by the work of Bronsted and Carlsen [66] and Gross and his coworkers [22]. Since then, many investigations regarding the fine structure and physicochemical properties of marine collagen have been performed. However, to the best of our knowledge, such extensive studies on the collagenous profile of sponge material have been conducted only on *C. reniformis* and *Ircinia* species [23,25,26,27,29]. In this context, the main purpose of the current study was the morphological characterization of various isolated collagenous materials (InSC, ICC, and SlC) from *A. cannabina* and *S. carnosus*, while further biochemical and biophysical characterization was undertaken only for the ICCs, given their relatively higher solubility and purity.

It has also been proven that in Demospongiae, collagen, constituting exclusively the intercellular organic framework, amounts to approximately 10% of the total organic matter [27,67]. In the present study, collagen content was experimentally calculated to amount for the 12.6% and 5.0% dry weight of *A. cannabina* and *S. carnosus*, respectively. The co-isolation of collagen with spicules is justified by the spicule formation procedure, generally accomplished by specialized cells that supply mineral ions or organic macromolecular particles, primarily consisting of proteins, carbohydrates, lipids, and seldom by nucleic acids [68].

Furthermore, the aforementioned characteristic insolubility has prevented the determination of the thermal behavior of sponge collagens. To our knowledge, only a few efforts have been made to determine their thermal behavior [25,69]. Our results corroborate to the existing knowledge that the thermal stability of marine collagens, which exhibit lower denaturation temperatures due to their lower content of imino acids, is generally lower than that of mammalian collagens. The low denaturation temperature of sponge collagen may also reflect the ambient temperature in which marine organisms live [70]. Moreover, the thermal stability of collagen is also directly correlated with the environmental and body temperatures of organisms [71].

Overall, the low denaturation temperature of sponge collagen observed in the present study enables gelatin extraction at lower temperature compared to mammalian gelatin, therefore providing an economic benefit for using marine sponges as a raw material of gelatin for the food industry [72].

All of our results point out that sponges contain collagen that retains its helical structure throughout the isolation procedure and all of its measured characteristics confirm the less crosslinked form, as verified by the IR, amino acid analysis, DSC, and CD data.

## 4. Materials and Methods

### 4.1. Animal Material

Specimens of *A. cannabina* were collected by SCUBA diving in Kea Island, Aegean Sea, Greece, at a depth of 15–20 m, whereas specimens of *S. carnosus* were collected by dredging at Kyllini Bay, Ionian Sea, Greece, at a depth of 50–70 m, and kept frozen until analyzed. Voucher specimens have been deposited at the animal collection of the Department of Pharmacognosy and Chemistry of Natural Products, National and Kapodistrian University of Athens (ATPH/MP0300 and ATPH/MP0106, respectively).

### 4.2. Chemicals

Tris(hydroxymethyl)aminomethane was from Mallinckrodt (Dublin, Ireland) and EDTA from Serva (Heidelberg, Germany). Urea and sodium carbonate were from Merck (Kenilworth, NJ, USA), while trypsin from bovine pancreas Type I, (~12,443 benzoyl l-arginine ethyl ester BAEE units/mg protein) was from Sigma (Darmstadt, Germany).

### 4.3. Isolation of Insoluble Collagen, Intercellular Collagen, and Spongin-Like Collagen

Sponge specimens were chopped and foreign inorganic and organic material was removed before washing with tap water. Before further processing the sponge tissues were immersed in EtOH for 24 h. InSC was isolated using an alkaline denaturing homogenization buffer (0.1 M Tris-HCl, pH 9.5, 0.01 M EDTA, 8 M urea, 0.1 M 2-mercaptoethanol), as previously described [24,26], whereas ICC and SlC were isolated using a trypsin-containing extraction buffer (0.1% trypsin in 0.1 M bicarbonate buffer, pH 8.0) [22,23]. All collagen samples were collected after centrifugation (at 20,000× *g* and 50,000× *g*, respectively) and lyophilization.

### 4.4. Removal of Spicules from Insoluble Collagen

In order to remove the siliceous spicules, InSC was treated with an HF solution (10% *v*/*v*) for 20 min at room temperature. The exact conditions were standardized using siliceous spicules isolated as previously described [73]. The material was rinsed with distilled-deionized water until pH ~ 6 was reached and the HF-treated spicule-free collagen (SF-InSC) was isolated by centrifugation at 12,000× *g* for 20 min and subsequently lyophilized.

### 4.5. Electron Microscopy

For SEM analysis lyophilized collagen samples were placed on stubs by using a double face adhesive tape, covered with a thin layer of gold using a Bal-tec SCD 004 sputter coater and examined under either a Cambridge Stereoscan 240 scanning electron microscope or a Philips Quanta Inspect (FEI Company) scanning electron microscope with a tungsten filament (25 kV).

For TEM analysis a small amount of sample dispersed in distilled-deionized water was placed on a Formvar-coated grid and stained with 2% aqueous solution of PTA (phosphotungstic acid hydrate; pH adjusted to 3.3 by using a solution of 1 N NaOH), which revealed the banding pattern of the fibrils, but not the whole periodicity. Each grid was examined under a Philips CM10 transmission electron microscope equipped with an Olympus Veleta digital camera.

### 4.6. Infrared Spectroscopy

IR spectra of lyophilized collagen samples were measured on a Bruker Tensor 27 FT-IR spectrometer using the attenuated total reflection (ATR) method, at room temperature, in the range of 500–4000 cm^−1^.

### 4.7. Amino Acid Analysis

The amino acid profile analysis was performed at TAMU Protein Chemistry Lab (College Station, TX, USA). Finely-ground ICC samples (30–40 mg) were used for liquid HCl (6 N) hydrolysis. Hydrolyzed proteins were derivatized pre-column with o-phthalaldehyde and 9-fluoromethyl-chloroformate prior to separation and quantitation by reverse phase HPLC. The component amino acids were then separated by HPLC (Agilent 1260), detected by UV (Agilent G1365D) or fluorometry (Agilent G1321B), and quantitated. All system control and data analysis was performed by Agilent Chemstation software. Values are the means of two independent experiments that did not differ by more than 2.9%.

### 4.8. Titration

Samples (70 mg) of freeze-dried material (SF-InSC) were dispersed in 7 mL distilled-deionized water by ultrasonication (GeneralSonic GS3) [26]. One sample was titrated with 0.1 N NaOH and the other with 0.1 N HCl. After each titrant addition, the suspension was stirred for 10 min at room temperature and subsequently the pH was recorded (Jenway 3310 pHmeter). A blank sample without collagen was titrated under the same conditions. The resulting pHs versus the amount of NaOH and HCl were plotted within the pH range from 2 to 12.

### 4.9. Differential Scanning Calorimetry

ICCs were initially dispersed in 0.01 M EDTA, pH 8.0 and the resulting suspension remained under stirring overnight at 8 °C. The collagens were collected after centrifugation at 13,000 rpm for 15 min. Subsequently, a 1% SDS (*w*/*v*) solution was used for the removal of entangled glycoconjugates [46]. Finally, the collagens were collected after centrifugation, washed exhaustively with distilled-deionized water, and lyophilized.

TMDSC measurements were performed by employing a MDSC 2920 calorimeter (TA Instruments, New Castle, DE, USA) under nitrogen flow (20 mL/min), using a heating rate of 2 °C/min, a temperature modulation amplitude of 0.318 °C every 60 s, and an empty pan as a reference. In such experiments the linear heating rate is superimposed by a sinusoidal temperature variation and it is, thus, possible to separate the total signal (corresponding to that of a conventional DSC) into two different components, corresponding to the reversible and the irreversible heat flows. The TMDSC profiles were obtained only on heating. Heat and temperature calibrations were performed by using indium as a standard. The enthalpic content (Δ*H*) of each transition was calculated from the area under each peak, while the transition temperature was taken at the center of each transition. For each experiment ~2 mg of lyophilized collagen, weighted with an accuracy of ± 0.01 mg, was hydrated with distilled-deionized water at a collagen/water ratio of 1:20 (*w*/*w*) and placed in sealed aluminum pans. The samples were then kept at 4 °C for 48 h before analysis.

### 4.10. Circular Dichroism Spectroscopy

The molecular conformation and denaturation temperature (T_d_) of ICCs, dissolved in distilled-deionized water to a concentration of 0.1 mg/mL, were assessed by CD spectroscopy using a Jasco J-715 circular dichroism spectropolarimeter equipped with a Peltier-type temperature control system (Jasco PTC-348Wi). CD spectra were recorded at 20 °C using a 0.1-cm path length quartz cell at 190–250 nm with a step size of 0.5 nm and a band width of 1.0 nm. Experiments were run in triplicate, and 10 scans for each spectrum were signal-averaged.

To determine the T_d_, the rotatory angle at a fixed wavelength of 221 nm, [*θ*]_221_, was recorded with heating from 15 to 50 °C at a rate of 1 °C/min. The collagen concentration was adjusted to 0.1 mg/mL and the temperature was controlled. The T_d_ was determined as the midpoint temperature between native-folded and completely unfolded forms. The mean molecular ellipticity (*θ*) was calculated using the equation [*θ*] = 10^−3^ *θ* M/LC (expressed in deg cm^2^ dmol^−1^), where *θ* is the measured ellipticity in degrees, L is the path length in mm, C is the concentration in mg/mL, and M is the average residue molecular weight of collagen equal to 91.2 [74].

## 5. Conclusions

In the present study, the collagenous content of the demosponges *A. cannabina* and *S. carnosus* was exhaustively examined. The insoluble, intercellular, and spongin-like collagens were isolated from *A. cannabina* and *S. carnosus*, representing 12.6%, 3.0%, and 42.8% dry weight for the former and 5.0%, 1.9%, and 21.8% dry weight for the latter sponge. SEM and TEM observations confirmed the characteristic fibrous structures, while IR spectroscopic analysis verified the characteristic absorption bands for proteins of the collagen class. Moreover, the acid–base properties of the insoluble collagen were investigated by titration, placing the isoelectric point approximately at pH 7. Marine sponge collagen, as compared to that derived from terrestrial animals and other marine collagen sources, has been reported to differentiate in its characteristics, such as amino acid composition, which consecutively affects collagen’s thermal behavior, isoelectric pH, solubility, and many other properties. In our case, the measured low imino acid content for the intercellular collagen, already reported being low in marine sources and even lower, specifically, in sponges, results in thermal stability comparable to that determined for collagen isolated from edible jellyfish and tropical fish. Indeed, the denaturation temperatures of the intercellular collagen isolated from *A. cannabina* and *S. carnosus* were determined by DSC studies at 25.4 °C and 32.9 °C, respectively, the first one being relatively lower than that reported for other marine organisms, while the second one being comparable to values observed for an array of collagens isolated from tropical fish. CD spectra indicated the existence of helical structures and the fact that the denaturation temperatures were dependent on the amount of imino acids. Marine collagen is considered as an equivalent, although safer, biomaterial than the one from terrestrial sources dominating the market nowadays. Our results suggest that the sponges *A. cannabina* and *S. carnosus* can be considered as an alternative source of collagen.

## Figures and Tables

**Table 1:** Collagen composition (*w*/*w* %) ^1^ of the sponges *A. cannabina* and *S. carnosus*.

Isolated Collagen	*A. cannabina*	*S. carnosus*
Insoluble collagen (InSC)	12.6	5.0
Intercellular collagen (ICC)	3.0	1.9
Spongin-like collagen (SlC)	42.8	21.8

^1^ Data are presented as the percent of sponge dry weight.

**Table 2:** Morphological characteristics (presented as means ± S.E.) of insoluble collagen (InSC), intracellular collagen (ICC) and spongin-like collagen (SlC) isolated from *A. cannabina* and *S. carnosus*.

Isolated Collagen	Period (nm) *n* = 10	Fibril Width (nm) *n* = 10
ICC from *A. cannabina*	31.29 ± 1.14 ^1^	18.74 ± 1.27 ^1^
ICC from *S. carnosus*	28.81 ± 1.73 ^2^	19.91 ± 1.65 ^1^
SlC from *S. carnosus*	26.60 ± 0.95 ^3^	24.10 ± 1.54 ^3^
InSC from *S. carnosus*		17.62 ± 2.91 ^1,4^

^1,2,3,4^ Data denoted by the same superscript are not significantly different (*p* > 0.05).

**Table 3:** IR spectra peak position and assignments for insoluble collagen before (InSC) and after (SF-InSC) spicules removal, intracellular collagen (ICC), and spongin-like collagen (SlC) isolated from *A. cannabina* and *S. carnosus*. For comparison reasons, the respective peaks for bovine collagen (BOC) [34] are also included.

Region	Peak Wavenumber (cm^−1^)								
	*A. cannabina*				*S. carnosus*				BOC
	InSC	SF-InSC	ICC	SlC	InSC	SF-InSC	ICC	SlC	
Amide A	3288	3279	3294	3286	3282	3282	3292	3287	3295
Amide B	2924	2924	2922	2926	2924	2922	2922	2923	2933
Amide I	1622	1627	1654	1639	1622	1628	1652	1647	1635
Amide II	1543	1529	1547	1539	1535	1527	1543	1543	1545
Amide III	1232	1226	1238	1222	1234	1230	1238	1232	1235
C-O stretch	1055	1059	1078		1074	1066	1078		
	1035	1037	1028	1028	1028	1031	1035	1001	

**Table 4:** Amino acid composition (residue/1000) of intercellular collagen (ICC) isolated from *A. cannabina* and *S. carnosus*.

Amino Acid	*A. cannabina*	*S. carnosus*	Amino Acid	*A. cannabina*	*S. carnosus*
Hyp	38	47	Met	21	11
Asx ^1^	100	94	Ile	37	24
Thr	56	56	Leu	62	48
Ser	63	57	Tyr	13	12
Glx ^2^	82	81	Phe	33	22
Pro	58	56	Hyl	6	6
Gly	257	295	Lys	15	15
Ala	72	89	His	6	4
Val	44	43	Arg	37	43
Total imino acids	96	103			

^1^ Asx: Asp + Asn. ^2^ Glx: Gln + Glu.
